# Relationship between the natural cessation time of umbilical cord pulsation in full-term newborns delivered vaginally and maternal-neonatal outcomes: a prospective cohort study

**DOI:** 10.1186/s12884-024-06444-9

**Published:** 2024-04-04

**Authors:** Ruijie Wu, Yuan Zhang, Jiaqi Chen, Tongchao Zhang, Xiaorong Yang, Xiangyu Xu, Mi Li, Dong Li, Xiaoyan Liu, Ming Lu

**Affiliations:** 1 School of Public Health, Cheeloo College of Medicine, Shandong University, Jinan, Shandong 250012 People’s Republic of China; 2 Clinical Epidemiology Unit, Qilu Hospital of Shandong University, Jinan, Shandong 250012 People’s Republic of China; 3 Clinical Research Center, Shandong University, Jinan, Shandong 250012 People’s Republic of China; 4 Department of Gynecology and Obstetrics, Qilu Hospital of Shandong University, Jinan, Shandong 250012 People’s Republic of China; 5 Department of Paediatrics, Qilu Hospital of Shandong University, Jinan, Shandong 250012 People’s Republic of China

**Keywords:** Vaginal delivery, Full-term newborns, Umbilical cord pulsation, Natural cessation, Maternal and infant outcomes

## Abstract

**Background:**

To analyze the impact of the time of natural cessation of the umbilical cord on maternal and infant outcomes in order to explore the time of clamping that would be beneficial to maternal and infant outcomes.

**Methods:**

The study was a cohort study and pregnant women who met the inclusion and exclusion criteria at the Obstetrics and Gynecology Department of Qilu Hospital of Shandong University from September 2020 to September 2021. Analysis using Kruskal-Wallis rank sum test, Pearson’s Chi-squared test, generalized linear mixed model (GLMM) and repeated measures ANOVA. If the difference between groups was statistically significant, the Bonferroni test was then performed. A two-sided test of *P* < 0.05 was considered statistically significant.

**Results:**

A total of 345 pregnants were included in this study. The subjects were divided into the ≤60 seconds group (*n* = 134), the 61–89 seconds group (*n* = 106) and the ≥90 seconds group (*n* = 105) according to the time of natural arrest of the umbilical cord. There was no statistically significant difference in the amount of postpartum hemorrhage and the need for iron, medication, or supplements in the postpartum period between the different cord spontaneous arrest time groups for mothers (*P* > 0.05). The weight of the newborns in the three groups was (3316.27 ± 356.70) g, (3387.26 ± 379.20) g, and (3455.52 ± 363.78) g, respectively, and the number of days of cord detachment was 12.00 (8.00, 15.75) days, 10.00 (7.00, 15.00) days and 9.00 (7.00, 13.00) days, respectively, as the time of natural cessation of the cord increased. The neonatal lymphocyte ratio, erythrocyte pressure, and hemoglobin reached a maximum in the 61–89 s group at (7.41 ± 2.16) %, (61.77 ± 8.17) % and (194.52 ± 25.84) g/L, respectively. Lower incidence of neonatal hyperbilirubinemia in the 61–89 s group compared to the ≥90s group 0 vs 4.8 (*P* < 0.05).

**Conclusions:**

In full-term singleton vaginal births, maternal and infant outcomes are better when waiting for 61–89 s after birth for the cord to stop pulsating naturally, suggesting that we can wait up to 90s for the cord to stop pulsating naturally, and if the cord does not stop pulsating after 90s, artificial weaning may be more beneficial to maternal and infant outcomes.

**Supplementary Information:**

The online version contains supplementary material available at 10.1186/s12884-024-06444-9.

## Introduction

After delivery, the newborn still shares blood circulation with the placenta and umbilical cord. The transfer of blood from the placenta to the newborn is a quite rapid stepwise progression and is almost finished in 3 minutes [1]. When the infant is delivered, about 30% of the blood is present in the placenta [2]. The blood flows into the newborn with the contraction of the umbilical artery and the uterus. The relationship between umbilical cord blood flow and umbilical cord pulsation is more complex. When the umbilical cord pulsation stops, the umbilical cord blood flow may not stop yet, and the blood flow may be still from mother to foetus [3]. If immediate or early cord clamping (ECC) is replied to, it may reduce the incidence of hyperbilirubinemia and polycythemia in newborns, and also reduce the requirement for phototherapy [4]. However, it may also increase the need for neonatal oxygen therapy and the risk of maternal postpartum hemorrhage [5]. Delayed cord clamping (DCC) usually defined as clamping the umbilical cord more than 60 second after delivery or when cord pulsation stops [6]. DCC may cause some benefits. It can reduce the incidence of anemia, iron deficiency, iron deficiency anemia, intraventricular hemorrhage (IVH), chronic lung disease and necrotizing enterocolitis. It could equally have a positive impact on the neurological and cognitive development of children aged 8 months and 12 months [7, 8]. It also improves the survival rate of preterm infants and reduces the risk of maternal anemia due to blood loss during the third stage of labor [9]. Potential disadvantages of DCC are the need for jaundice treatment and maternal blood transfusion [6, 8]. Therefore, there are advantages and disadvantages to early or delayed cord clamping for mothers and infants (both preterm and term), and there is still considerable international debate about the best time to clamp the umbilical cord.

The American College of Obstetricians and Gynecologists (ACOG) recommends delayed umbilical cord clamping in full-term neonates and preterm infants for at least 30–60 seconds after birth [10]. The World Health Organization (WHO) recommends that the umbilical cord should be clamped between 1 and 3 minutes after birth as part of the active management of the third stage of labor (AMTSL), in order to improve maternal and neonatal outcomes unless the baby has special circumstances such as neonatal asphyxia and the need for resuscitation [11]. National Institute for Health and Care Excellence (NICE) recommends if there is no concern about the integrity of the umbilical cord or if the baby’s heart rate is below 60 beats per minute and is not getting faster, it is best to clamp the cord 1–5 minutes after delivery [12].

Whether to clamp the umbilical cord early or delayed has always been a controversial topic [13]. Although a large number of studies have shown the positive impact of delayed cord clamping on mother and baby, most have not focused on the impact of the time to natural cessation of cord pulsation on maternal and infant outcomes. And there is still a shortfall in guiding the recommended optimal time to clamp the cord. In our study, artificial clipping was performed when the umbilical cord pulsation naturally stopped, disregarding the complex relationship between subsequent umbilical blood flow and umbilical cord pulsation. We aimed to compare the relationship of different times of umbilical cord disconnection with the outcomes of mothers and infants.

## Methods

### Study design and participants

We recruited study participants (women who delivered full-term newborns) for a prospective cohort study at the Department of Gynecology and Obstetrics, Qilu Hospital, Shandong University between September 2020 and September 2021. Full-term infants were defined as gestational age between 37 and 42 weeks. Inclusion criteria: (1) pregnant women age from 18 to 35 years; (2) 37 weeks gestation; (3) vaginal delivery, including lateral episiotomy; (4) participants who voluntarily participate in the study will obtain written informed consent before participation in the study. Exclusion criteria: (1) pregnant women diagnosed with COVID-19; (2)pregnant women with internal and surgical comorbidities; (3) maternal anemia; (4) multiple pregnancies; (5) ABO hemolysis, RH hemolysis; (6) full-term newborns are found to have anomalies and the anomalies affect bilirubin metabolism, such as congenital anal atresia and congenital biliary atresia; (7) pneumonia, hemolytic disease of term newborns and other diseases affecting bilirubin metabolism during the follow-up of term newborns; (8) term small for gestational age (term-SGA); (9) abnormalities on examination after delivery of the placenta, such as placenta praevia, placenta abruptio, placenta rotunda and anterior vascular hemorrhage; (10) stillbirth; (11) postnatal full-term newborn Apgar score ≤ 7 at 1 minute.

### The natural cessation time of umbilical cord pulsation and grouping

The interval between the delivery of the baby and the cessation of the pulse of the chord is referred to as the time of cessation of the pulsation of the cord. After birth, the baby was placed between the mother’s knees, wrapped in a sterile towel to keep warm, and the midwife uses her palm to detect the pulse of the umbilical cord close to the baby’s end, while the mobile midwife uses a movement stopwatch to time the movement to the second. When the umbilical artery stops pulsing, the umbilical cord is cut and ligated immediately.

Based on recommendations from the World Health Organization and other agencies, and combined with data collected in the study, participants were divided into three groups according to the duration of umbilical cord arrest: ≤60 seconds, 61–89 seconds, and ≥ 90 seconds.

### Study variables

Participants in the study were surveyed using convenience sampling, a non-probability sample method. The variables we collected can be summarized in four parts, the first being demographic information collected after admission to the maternity ward, such as mother’s name, age, occupation, education, ethnicity, BMI (pre-pregnancy and pre-natal), blood type, smoking and alcohol consumption. The second part is clinical information, such as maternal blood count (hemoglobin, red blood cell pressure, etc.) within 1 day before delivery, the cord blood and the total nucleated cells (TNCs) after the umbilical cord is severed. The third section is maternal outcomes, such as maternal postpartum hemorrhage (recorded separately 2 and 24 hours after delivery), blood transfusions (24 hours after delivery), and medication (iron, other drugs or health supplements) for 42 days after delivery. The fourth section is neonatal outcomes, such as blood gas analysis of the umbilical cord artery and vein after delivery (this indicator is taken by the midwife on the delivery team immediately after delivery and blood gas analysis is performed), birth weight and sex of the newborn obtained 2 hours after delivery, blood bilirubin (24 hours, 48 hours, 72 hours, 5 days, 10 days, 42 days after delivery of the newborn). Newborn blood bilirubin was measured using a JH20-1C transcutaneous yellow pox meter and averaged at four points on the forehead, brow, corner of the eye and forehead.

### Statistical analysis

The main observation index is the time of cessation of umbilical cord pulsation, according to previous studies [13], the time of umbilical cord pulsation is (120.1 ± 85.9) seconds, the expected error is not more than 10, take α = 0.05 to substitute into the formula:

$$\textrm{n}={\left[\frac{u_{1-\alpha /2}\sigma }{\delta}\right]}^2,$$

and calculate *n* = 284, considering the lost to follow-up and other factors, set a 5% loss to follow-up rate, the number is at least 300 pregnant women.

Descriptive statistics were used for the clinical characteristics of the population. Data for normal continuous variables are presented as mean and standard deviation (SD), and skewed continuous variables are presented as median and interquartile range (IQR); categorical variables are described by frequency and percentage. For maternal and infant outcomes in the different cord clamping groups, the Kruskal-Wallis rank sum test was used for continuous variables to assess the statistical significance of differences; the Pearson’s Chi-squared test was used for categorical information; those with overall differences were then compared between two groups. For the six time points of 24 hours, 48 hours, 72 hours, 5 days, 10 days and 42 days after delivery, we used a generalized linear mixed model (GLMM) to compare the differences between the neonatal outcomes of the different subgroups, using repeated measures of categorical information such as whether the neonate had hyperbilirubinaemia, was taking health supplements or medication. Similarly, for neonatal blood bilirubin at the six time points above, we used repeated measures ANOVA to compare differences between groups and between time points, and then carried out Tukey post-hoc tests if the differences were statistically significant. A value of *P* < 0.05 for a two-sided test is considered statistically significant. All analyses were performed in R program (Version 4.2.2, R core team, 2022) and IBM SPSS Statistics 25.

## Results

### Overview

**Fig. 1:**
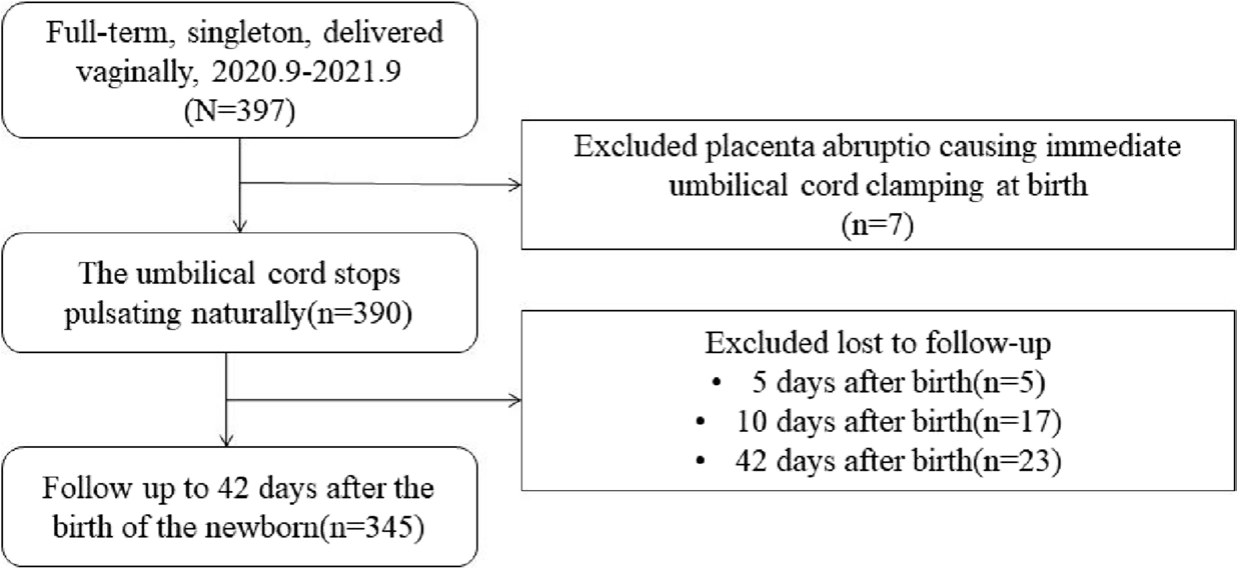
Participant flowchart

Between September 2020 to September 2021, 397 pregnant women met the inclusion and exclusion criteria. Of these, 7 were excluded for immediate umbilical cord clamping due to placental abruption after delivery., and 45 were lost to follow-up. Finally, 345 participants who met the criteria and had complete data were analyzed (Fig. 1).

### The natural cessation time of umbilical cord pulsation

**Fig. 2:**
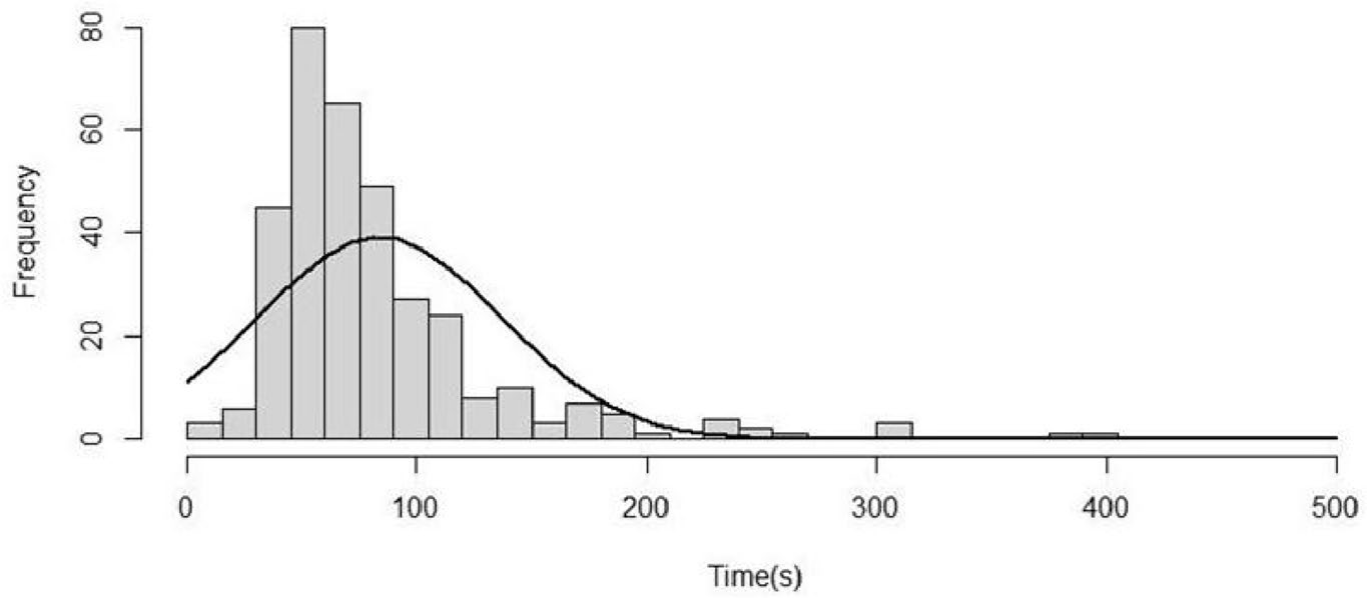
Distribution of time to natural cessation of umbilical cord pulsation in the study population

In this study, a total of 345 participants were included. They were divided into three groups based on the cord-clamping time, with 134, 106 and 105 participants in each group from shortest to longest time respectively. The age of maternal is 31.0 ± 4.0 years. The range of time of umbilical cord pulsation cessation is 10.00 ~ 403.00 seconds. The median time is 68.00 seconds (IQR: 54.00–95.00).A Shapiro-Wilk normality test for the natural cessation time of umbilical cord pulsation revealed a positively skewed distribution of the data (*P* < 0.05, Kurtosis = 12.01, Skewness = 2.56) (Fig. 2).

### Baseline characteristics for maternal

Basic characteristics of maternal are presented in Table 1. In terms of occupation and chronic illness, there were statistically significant differences between the three groups. In the three groups, 16.4, 13.2 and 4.8% of the mothers had chronic diseases, respectively, according to the shortest to longest duration of spontaneous cessation of the cord’s pulsation. Whereas, there were no differences between the groups concerning age, age at menarche, BMI, education, gestational age, lifestyle habits (exposure to smoking and alcohol), drugs or health supplements during pregnancy (drugs are mainly iron, and health supplements are mainly vitamins and DHA), oxygen inhalation after 36 weeks of pregnancy (on medical advice or on the mother’s own will). There was also no statistically significant difference between the groups with or without oxytocin and glucose solution without electrolytes before delivery and the time of umbilical clamping (*P* > 0.05)(Table 1).

**Table 1:** Baseline demographic and clinical characteristics of maternal

Characteristic	≤60s (*n* = 134)	61–89 s (*n* = 106)	≥90s (*n* = 105)	*P*
Age (year)	30.99 ± 3.98	30.95 ± 4.23	30.93 ± 3.86	> 0.990
Age at menarche (year)				0.890
≤ 14	115(85.8)	93(87.7)	90(85.7)	
> 14	19(14.2)	13(12.3)	15(14.3)	
BMI (kg/m^2^)				
Pre-pregnancy	21.83 ± 3.69	21.91 ± 2.75	21.65 ± 3.16	0.560
Antenatal	26.75 ± 4.19	26.83 ± 3.69	26.98 ± 3.28	0.930
Occupation				0.036^#^
Civil servant	23 (17.0)	34 (32.0)	19 (18.0)	
Professional and technical staff	50 (37.0)	37 (35.0)	45 (43.0)	
Other	61 (46.0)	35 (33.0)	41 (39.0)	
Education				0.880
High level	31 (23.0)	21 (20.0)	24 (23.0)	
Middle level	61 (46.0)	55 (52.0)	48 (46.0)	
Low level	42 (31.0)	30 (28.0)	33 (31.0)	
Smoking during pregnancy				0.640
Yes	2 (1.0)	0 (0.0)	1 (1.0)	
No	132 (99.0)	106 (100.0)	104 (99.0)	
Passive smoking before pregnancy				0.370
Yes	28 (21.0)	15 (14.0)	21 (20.0)	
No	106 (79.0)	91 (86.0)	84 (80.0)	
Drinking alcohol before pregnancy				0.670
Yes	2 (1.0)	1 (1.80)	0 (0.0)	
No	132 (99.0)	105 (98.20)	105 (100.0)	
History of chronic illness				0.019^*^
Yes	22 (16.4)	14 (13.2)	5 (4.8)	
No	112 (83.6)	92 (86.8)	100 (95.2)	
Drugs or health supplements during pregnancy				0.840
Yes	109 (81.0)	87 (82.0)	83 (79.0)	
No	25 (19.0)	19 (18.0)	22 (21.0)	
Oxygen inhalation after 36 weeks of pregnancy				0.220
Yes	23 (17.2)	10 (9.0)	14 (13.0)	
No	111 (82.8)	96 (91.0)	91 (87.0)	
Pregnant mother’s blood type (ABO)				0.100
A	27 (20.0)	28 (26.0)	31 (30.0)	
B	48 (36.0)	31 (29.0)	40 (38.0)	
AB	16 (12.0)	16 (15.0)	4 (4.0)	
O	43 (32.0)	31 (29.0)	30 (28.0)	
Pregnant mother’s blood type (RH)				0.600
RH (+)	132 (99.0)	105 (99.0)	102 (97.0)	
RH (−)	2 (1.0)	1 (1.0)	3 (3.0)	
Spouse’s blood type (ABO)				0.091
A	20 (15.0)	19 (18.0)	8 (8.0)	
B	75 (56.0)	50 (47.0)	54 (51.0)	
AB	12 (9.0)	10 (10.0)	19 (18.0)	
O	27 (20.0)	27 (25.0)	24 (23.0)	
Spouse’s blood type (RH)				> 0.990
RH (+)	133 (99.0)	106 (100.0)	105 (100.0)	
RH (−)	1 (1.0)	0 (0.0)	0 (0.0)	
Gestation				0.460
1	60 (45.0)	43 (40.60)	43 (41.0)	
2	45 (34.0)	42 (39.60)	32 (30.0)	
≥ 3	29 (22.0)	21 (19.80)	30 (29.0)	
Production				0.650
1	81 (60.0)	66 (62.0)	59 (56.0)	
≥ 2	53 (40.0)	40 (38.0)	46 (44.0)	
Live				0.720
1	82 (61.0)	66 (62.0)	60 (57.0)	
≥ 2	52 (39.0)	40 (38.0)	45 (43.0)	
Abortion				0.120
0	91 (68.0)	70 (66.0)	64 (61.0)	
1	28 (21.0)	20 (19.0)	33 (31.0)	
≥ 2	15 (11.0)	16 (15.0)	8 (8.0)	
Gestational age (days)	276.46 ± 7.03	276.85 ± 6.98	277.49 ± 6.69	0.540
Oxytocin				0.820
Yes	47 (34.0)	41 (38.0)	40 (39.0)	
No	87 (66.0)	65 (62.0)	65 (61.0)	
Glucose solution without electrolytes use				0.470
Yes	5 (4.0)	2 (2.0)	5 (5.0)	
No	129 (96.0)	104 (98.0)	100 (95.0)	
Assisted reproductive technologies				0.631
Yes	1(0.8)	1(0.9)	0(0.0)	
No	133(99.2)	105(99.1)	105(100.0)	

Normal continuous variables: mean ± SD. Skewed continuous variables: median (IQR). Categorical variables: n, %. SD, Standard Deviation. IQR, Interquartile Range.

^#^The significance values were adjusted for multiple testing by Bonferroni correction and statistically significant differences were found between ≤60s group and 61–89 s group in civil servant

^*^The significance values were adjusted for multiple testing by Bonferroni correction and statistically significant differences were found between ≤60s group and ≥ 90s group in Yes and No

### Maternal blood routine examination

Differences in maternal PCT (Plateletcrit) within 7 days antenatal were statistically significant in different neonatal cord natural clamping of pulsation time groups. Differences in routine hematological indices at 24 hours postpartum were not statistically significant in the different subgroups (Supplemental Table 1).

### Indicators related to the postnatal period of maternal

There were no statistically significant differences between the three groups in postnatal indicators, such as the amount of bleeding and medication taken by the mothers (*P* > 0.05) (Table 2).

**Table 2:** Postnatal indicators in mothers with different times of natural cessation of umbilical cord pulsation

Characteristic	≤60s (*n* = 134)	61–89 s (*n* = 106)	≥90s (*n* = 105)	*P*
Bleeding (ml)				
2 hours after delivery	230.00 (210.00, 259.00)	230.00 (210.00, 257.75)	220.00 (200.00, 250.00)	0.280
24 hours after delivery	370.00 (320.00, 441.00)	365.00 (315.75, 420.00)	375.00 (335.00, 440.00)	0.600
Iron within 42 days after delivery				0.960
Yes	14 (10.4)	11 (10.4)	12 (11.4)	
No	120 (89.6)	95 (89.6)	93 (88.6)	
Medication or other health supplements within 42 days of delivery				0.380
Yes	30 (22.4)	20 (18.9)	16 (15.2)	
No	104 (77.6)	86 (81.1)	89 (84.8)	

Normal continuous variables: mean ± SD. Skewed continuous variables: median (IQR). Categorical variables: n, %. SD, Standard Deviation. IQR, Interquartile Range

### Characteristics of newborns after grouping according to the time of natural cessation of umbilical cord pulsation

The gender distribution of newborns was balanced among the three groups, with a male-to-female ratio of 75 (55.9%) vs 59 (44.1%), 62 (58.5%) vs 44 (41.5) %, and 52 (49.5%) vs 53 (50.5%) respectively. There was a statistically significant difference in the birth weight of newborns in the different time of umbilical cord clamping groups. As weight increased, the time to natural cessation of the umbilical cord pulsation was correspondingly longer in newborns (*P* < 0.05). Similarly, there was a statistically significant difference between the three groups in the number of days the umbilical cord was detached in newborns. The number of days of umbilical cord detachment decreased with increasing time of cord clamping (*P* < 0.05). As the time of natural cessation of umbilical cord pulsation increased, there were no statistically significant differences in cord blood nucleated cell counts and cord blood pH among the three groups, except for the differences in cord and placenta related indices among the three groups (*P <* 0.05). All newborns had Apgar scores of 10 at 1, 5 and 10 minutes (Table 3).

**Table 3:** Demographic and clinical characteristics of infants at different times of umbilical cord clamping

Characteristic	≤60s (*n* = 134)	61–89 s (*n* = 106)	≥90s (*n* = 105)	*P*
Gender				0.400
Male	75 (55.9)	62 (58.5)	52 (49.5)	
Female	59 (44.1)	44 (41.5)	53 (50.5)	
Length(cm)	50.49 ± 2.15	50.59 ± 2.00	50.91 ± 2.02	0.210
Weight(g)	3316.27 ± 356.70	3387.26 ± 379.20	3455.52 ± 363.78	0.037^a^
Umbilical cord abscission	12.00 (8.00, 15.75)	10.00 (7.00, 15.00)	9.00 (7.00, 13.00)	0.008^a^
Cord blood				
Mass (g)	32.00 (23.40, 43.82)	32.00 (21.92, 41.42)	34.20 (24.50, 45.60)	0.330
Volume (ml)	56.00 (46.00, 68.00)	56.00 (46.00, 70.00)	60.00 (48.00, 68.00)	0.370
Total nucleated cells	2.83 (1.45, 4.34)	3.11 (1.81, 5.80)	3.55 (2.00, 5.04)	0.040
Placenta				
Weight (g)	536.32 ± 104.23	536.84 ± 106.55	555.15 ± 98.23	0.250
Volume (cm^3^)	758.21 ± 156.00	746.72 ± 164.68	753.49 ± 154.49	0.890
Umbilical cord length (cm)	60.28 ± 13.15	59.28 ± 9.05	58.62 ± 11.31	0.930
pH	7.33 ± 0.07	7.30 ± 0.07	7.30 ± 0.06	0.010^a^
pO_2_	27.80(21.93, 32.73)	25.05(21.03, 29.88)	27.00(20.43, 31.33)	0.146
pCO_2_	42.31 ± 9.25	44.55 ± 9.46	44.20 ± 7.64	0.111
Apgar 1 minute				–
10	134 (100%)	106 (100%)	105 (100%)	
Apgar 5 minutes				–
10	134 (100%)	106 (100%)	105 (100%)	
Apgar 10 minutes				–
10	134 (100%)	106 (100%)	105 (100%)	
Symptomatic polycythemia				0.210
Yes	10 (7.5)	3 (2.8)	4 (3.8)	
No	124 (92.5)	103 (97.2)	101 (96.2)	

Normal continuous variables: mean ± SD. Skewed continuous variables: median (IQR). Categorical variables: n, %. SD, Standard Deviation. IQR, Interquartile Range.

^a^The significance values were adjusted for multiple testing by Bonferroni correction and statistically significant differences were found between ≤60s group and ≥ 90s group

### Infants blood routine examination

Among the routine blood indicators of newborns within 24 hours of birth, there were statistically significant differences in WBC, RBC, LYM, PCV and HGB among the subgroups at different times of umbilical cord clamping. Furthermore, LYM (7.41 ± 2.16%), PCV (61.77 ± 8.17%) and HGB (194.52 ± 25.84 g/L) all reached their maximum in the 61–89 s group (Table 4).

**Table 4:** Blood count within 24 hours after delivery of the newborn

Characteristic	≤60s (*n* = 134)	61–89 s (*n* = 106)	≥90s (*n* = 105)	*P*
WBC (× 10^9^/L)	21.03 ± 7.92	20.80 ± 4.85	19.44 ± 5.90	0.043^b^
NEU (×10^9^/L)	53.22 ± 9.01	54.40 ± 8.23	54.54 ± 9.51	0.120
LYM (×10^9^/L)	36.94 ± 8.85	36.11 ± 7.87	35.89 ± 9.94	0.180
EOS (×10^9^/L)	2.60 (1.75, 3.20)	2.55 (1.90, 3.70)	2.80 (2.00, 3.50)	0.370
BAS (×10^9^/L)	0.30 (0.10, 0.40)	0.30 (0.20, 0.50)	0.30 (0.20, 0.40)	0.580
MON (×10^9^/L)	6.50 ± 1.69	6.12 ± 1.57	6.21 ± 1.63	0.062
PLT (×10^9^/L)	265.04 ± 81.04	266.10 ± 74.61	244.37 ± 82.01	0.200
RBC (× 10^12^/L)	5.41 ± 1.04	5.40 ± 0.81	5.31 ± 0.64	0.045^a^
NEU (%)	11.20 ± 4.76	11.41 ± 3.62	10.67 ± 3.69	0.280
LYM (%)	7.39 ± 1.98	7.41 ± 2.16	6.91 ± 2.85	0.006^a,b^
EOS (%)	0.51 (0.34, 0.69)	0.50 (0.38, 0.75)	0.50 (0.37, 0.65)	0.650
BAS (%)	0.05 (0.02, 0.09)	0.06 (0.03, 0.09)	0.05 (0.03, 0.08)	0.190
MON (%)	1.29 (1.04, 1.61)	1.24 (0.97, 1.53)	1.15 (0.94, 1.42)	0.066
PCV/Hct (%)	61.28 ± 11.10	61.77 ± 8.17	59.91 ± 6.89	0.007^a^
HGB (g/L)	190.26 ± 44.50	194.52 ± 25.84	187.88 ± 27.17	0.010^a^
MCV (fL)	111.71 ± 13.69	111.82 ± 14.88	110.81 ± 14.92	0.480
MCH (pg)	35.90 ± 1.72	35.94 ± 1.58	35.70 ± 1.53	0.490
MCHC (g/L)	309.45 ± 48.00	312.71 ± 32.76	316.24 ± 11.78	0.510
RDW (%)	16.55 ± 4.25	16.71 ± 4.53	16.74 ± 4.55	0.920
PDW (fL)	16.60 (16.20, 17.00)	16.60 (16.30, 17.00)	16.60 (16.40, 16.90)	0.780
MPV (fL)	9.50 (9.00, 9.90)	9.40 (8.93, 9.80)	9.40 (8.90, 9.90)	0.910
PCT (%)	0.26 (0.21, 0.31)	0.25 (0.22, 0.30)	0.26 (0.19, 0.30)	0.540

*IQR* Interquartile Range, *SD* Standard Deviation, *WBC* White Blood Cell Count, *NEU* Neutrophil, *LYM* Lymphocyte, *EOS* Eosinophil, *BAS* Basophils, *MON* Monocytes, *PLT* Platelet Count, *RBC* Red Blood Cell Count, *PCV* Packed Cell Volume, *HGB* Hemoglobin, *MCV* Mean Corpuscular Volume, *MCH* Mean Corpuscular Hemoglobin, *MCHC* Mean Corpuscular Hemoglobin Concentration, *RDW* Red Cell Distribution Width, *PDW* Platelet Distribution Width, *MPV* Mean Platelet Volume, *PCT* Plateletcrit

^a^The significance values were adjusted for multiple testing by Bonferroni correction and statistically significant differences were found between ≤60s group and ≥ 90s group

^b^The significance values were adjusted for multiple testing by Bonferroni correction and statistically significant differences were found between 61-89s group and ≥ 90s group

### Neonatal bilirubin and the incidence of neonatal hyperbilirubinemia

Bilirubin concentrations as detected by transcutaneous measurements at six time points: 24 hours, 48 hours, 72 hours, 5 days, 10 days and 42 days after the birth of the newborn showed statistically significant differences at different time points of measurement(*P* < 0.05), and the differences were not statistically significant in different subgroups of time to natural cessation of umbilical cord pulsation. Differences in the interaction of measurement time points and grouping factors were also not statistically significant (*P* > 0.05)(Supplemental Table 2). The Bonferroni post-hoc test showed a statistically significant difference between the ≤60s and ≥ 90s groups, with the ≥90s being higher than the ≤60s group at all different time points of measurement. Similarly, there was a statistically significant difference between the six measurement time points, with the maximum transcutaneous bilirubin reaching 5 days after birth in newborns (Supplemental Fig. 1). The difference in the incidence of neonatal hyperbilirubinemia on the tenth day of life was statistically significant between the three groups at 10 days after birth, with multiple comparisons revealing a statistically significant difference between the 61–89 seconds (0.0%) and ≥ 90 seconds (4.8%) groups (Table 5).

**Table 5:** Incidence of hyperbilirubinemia in neonates at six time points in groups with different times of cessation of cord pulsation after birth

Characteristic	≤60s (*n* = 134)	61–89 s (*n* = 106)	≥90s (*n* = 105)	*P*
Time (after birth), (n, %)				
24 hours				0.380
Yes	4(3.0)	5(4.7)	7(6.7)	
No	130(97.0)	101(95.3)	98(93.3)	
48 hours				0.150
Yes	1(0.7)	0	3(2.9)	
No	133(99.3)	106(100.0)	102(97.1)	
72 hours				0.320
Yes	5(3.7)	1(0.9)	2(1.9)	
No	129(96.3)	105(99.1)	103(98.1)	
5 days				0.390
Yes	5(3.7)	5(4.7)	8(7.6)	
No	129(96.3)	101(95.3)	97(92.4)	
10 days				0.020^b^
Yes	1(0.7)	0	5(4.8)	
No	133(99.3)	106(100.0)	100(95.2)	
42 days				> 0.990
Yes	1(0.7)	1(0.9)	0	
No	133(99.3)	105(99.1)	105(100.0)	

^b^The significance values were adjusted for multiple testing by Bonferroni correction and statistically significant differences were found between 61-89s  group and ≥ 90s group

### Use of health products or medicines for newborns

The study revealed that group of ≤60s had a higher utilization for health supplements (product packaging printed on the “health food” that is health supplements) or medication compared to group of 61–89s, with the sixth time point serving as the baseline. There was a notable statistical difference in the utilization for health supplements or medication between the two groups, with the difference becoming significant from the second time point onwards (*P* < 0.001). Additionally, as the observation period increased, the utilization for health supplements or medication among newborns also increased. Similarly, the need was higher in group of 61–89s compared to group of ≥90s (Supplemental Table 3, Supplemental Table 4).

## Discussion

The aim of the prospective cohort study was to compare the effects of different times of natural cord cessation on maternal and infant outcomes, in order to explore the most beneficial time of cord clamping for maternal and infant outcomes.

The time of natural cessation of umbilical cord pulsation may be related to the influence of factors, such as cord length and cord hemodynamics [3, 14]. In our study, neonatal umbilical cords stopped pulsing naturally at the time range of 10.00–403.00s, with a median duration of 68.00s (IQR: 54.00–95.00). Although most full-term neonates’ umbilical cord pulsing stop within 2 min of birth, there is study suggesting that the median duration of umbilical cord pulsing is 213.00s (IQR: 120.00–420.00) [15]. There is also study suggesting that the natural cessation of umbilical cord pulsation in neonates may be extraordinarily prolonged (the mean clamping time in the DCC group was 305 s) [16].

The present study showed that the timing of cord arrest was not affected by the mother’s blood count, including hemoglobin, within 7 days prior to delivery and 24 hours after delivery. Routine blood count is one of the most important indicators for assessing the health status of the mother and baby. Although they can provide information on the health status of the mother and baby, they are not the only factor in determining the time of umbilical cord breakage and the outcome of the mother and baby. In some cases, routine blood indicators may affect the timing of cord cutting and maternal and infant outcomes. Delayed umbilical cord clamping needs to be carefully considered and monitored in cases of maternal anemia or other risk factors associated with anemia [17]. Therefore, special attention should be paid to maternal hemoglobin levels and anemia status when considering the timing of umbilical cord cutting.

Postpartum hemorrhage (PPH) is the leading cause of maternal death in low-income countries, accounting for almost a quarter of all maternal deaths worldwide. PPH is defined as bleeding≥500 ml within 24 hours after the fetus is delivered [18]. In general, there is concern that the prolonged wound closure in the DCC group leads to a longer operative time and thus increased maternal blood loss. However, the study shows that the time of natural arrest of the umbilical cord in newborns does not have an effect on the amount of maternal postpartum hemorrhage (PPH). A meta-analysis by Judith Gomersall, MCom et al. that included 33 studies came to a similarly conclusion that early or late umbilical cord clamping may have little effect on maternal complications, including PPH (≥500 ml) [19].

For newborns, there may be a correlation between the weight of the newborn, the time when the umbilical cord stops pulsating naturally and the time when the newborn’s umbilical cord falls off. It may be that the better the mother’s health, the better the nutrients and survival conditions the newborn receives in the womb, resulting in a heavier newborn. The mother’s well-conditioned body may support the pulsation of the umbilical cord for longer. In addition, the care of the newborn such as the length of time it takes for the cord to fall off may also vary according to the health of the mother and the weight of the fetus, with the greater birth weight of the newborn, the quicker the cord falls off.

According to a review, no significant differences were observed between early and delayed cord clamping in terms of Apgar scores below 7 at 5 minutes [6]. This is consistent with the findings of our study. However, a study by Michael Fogarty et al. showed that delayed clamping reduced the incidence of 1-minute low Apgar scores in preterm infants [20]. Demonstrate that delayed cord clamping does not adversely affect postnatal Apgar scores, at least in newborns. A number of systematic reviews and meta-analyses have similarly reported that delayed cord clamping (DCC) may be more beneficial for preterm infants, such as reducing the chance of intraventricular hemorrhage, improving hemodynamic outcomes, reducing the need for blood transfusions, reducing hospital mortality, and reduced incidence of low Apgar scores within 1 minute [20, 21]. Overall, all of the above studies may provide evidence to demonstrate that delayed cord locking does not adversely affect postnatal Apgar scores, at least in term newborns.

Anemia is one of the most critical factors contributing to neonatal and infant mortality in developing countries [22]. Previous studies of cord clamping have shown beneficial effect of DCC on hemoglobin (Hb) at birth or at different follow-up times in term neonates and demonstrated reduction in anemia. A randomized controlled trial of expected low birthweight newborns in South Africa showed that hemoglobin levels were significantly higher in the DCC (2–3 min after birth) group at 24 hours after birth, but the difference disappeared at 2 months after birth [23]. Meanwhile, a randomized controlled trial in a highly malarious rural area of Zambia found that infants in the ICC group had a faster decline in Hb levels than the DCC group throughout the observation period, and this difference disappeared by 6 months of age [16]. The hematological benefit of DCC disappears after 4 months and may be related to the iron content of the infant’s body, partly due to increased iron stores during growth and partly due to the infant losing more iron through the gastrointestinal tract during diarrhoea or during the feeding of whole milk [24]. The study found statistically significant differences in hemoglobin and erythrocyte pressure among three groups within 24 hours of birth, with a maximum in the 61–89 second group. This suggests that waiting for the umbilical cord to stop beating naturally after delivery or disconnecting it at 61–89 seconds may be beneficial to the newborn.

Bilirubin is an important cytoprotective agent for brain tissue, enhancing its antioxidant defenses to a certain extent. Higher levels of bilirubin within the normal range can provide protective antioxidant effects to the developing brain [25]. A retrospective cohort study of 424 newborns showed that a delayed umbilical weaning regimen for term newborns was associated with significantly higher mean transcutaneous bilirubin levels [26]. This is consistent with our findings. Results of a randomized controlled trial including 73 study participants showed no increase in adverse effects such as hyperbilirubinemia or symptomatic polycythemia in infants who received DCC (≥5 minutes) between 24 and 48 hours [4].

Instead of randomizing the pregnants to ECC or DCC groups based on artificial umbilical cord disconnection in most of the randomized clinical trials, the present study explored the more favourable umbilical cord disconnection time for maternal and infant outcomes by waiting for the umbilical cord to stop pulsing naturally before disconnecting the umbilical cord, which is an issue that has been paid less attention to in the published studies. Additionally, we followed up some of the outcomes, and the results of the present study may provide new evidences to support the optimal umbilical cord disconnection time for newborns born full-term via vaginal delivery. This study has several limitations. Firstly, the study only included newborns born at term in singleton pregnancies delivered vaginally, so the findings may not be generalizable to multiple pregnancies, preterm births and cesarean sections, and further studies are needed to assess the impact of delayed umbilical cord clamping on maternal and infant outcomes in these circumstances. Secondly, the study did not follow up the mother and newborn for a longer period of time to examine the long-term effects of the time to spontaneous cord arrest on maternal and infant outcomes.

## Conclusions

Prolonged cessation of the natural pulsation of the umbilical cord may lead to increased hemoglobin levels in the newborn, but does not increase the risk of postnatal hemorrhage in the mother. It is recommended that the umbilical cord is cut within 61–89 seconds of delivery as this may be more beneficial to the health of the newborn and the recovery of the mother.

### Supplementary Information

**Supplementary Material 1.**

**Supplementary Material 2.**

## Data Availability

The datasets used and analyzed during the current study are available from the corresponding author on reasonable request.

## Abbreviations

ECC: Early cord clamping
PPH: Postpartum hemorrhage
DCC: Delayed cord clamping
IVH: Intraventricular hemorrhage
ACOG: The American College of Obstetricians and Gynecologists
WHO: The World Health Organization
AMTSL: The active management of the third stage of labor
NICE: National Institute for Health and Care Excellence
SD: Standard deviation
IQR: Interquartile range
GLMM: Generalized linear mixed model
