# Supplementary figures and images for: Relationship between the natural cessation time of umbilical cord pulsation in full-term newborns delivered vaginally and maternal-neonatal outcomes: a prospective cohort study
Source: BMC Pregnancy Childbirth. 2024 Apr 4;24:236. doi: 10.1186/s12884-024-06444-9 (PMC10993427; doi:10.1186/s12884-024-06444-9)

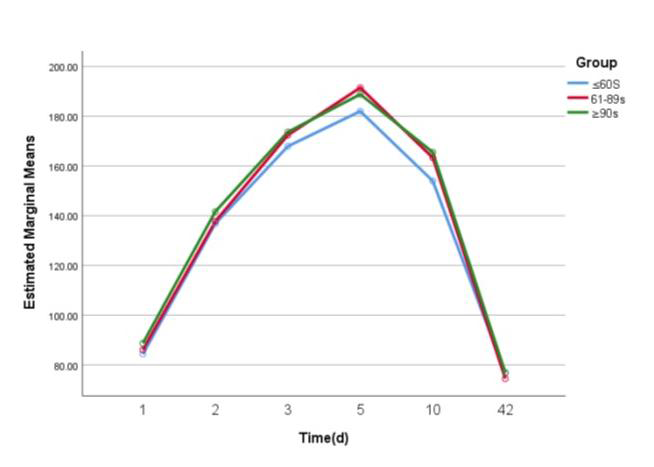

Supplement: Supplementary file 1 — Supplementary Material 1. [file 12884_2024_6444_MOESM1_ESM.tif]
