# Supplementary material for: Relationship between the natural cessation time of umbilical cord pulsation in full-term newborns delivered vaginally and maternal-neonatal outcomes: a prospective cohort study
Source: BMC Pregnancy Childbirth. 2024 Apr 4;24:236. doi: 10.1186/s12884-024-06444-9 (PMC10993427; doi:10.1186/s12884-024-06444-9)
Supplement: Supplementary file 2 — Supplementary Material 2. [file 12884_2024_6444_MOESM2_ESM.docx]

**Supplemental table1** Maternal blood count within 7 days before r delivery and 24 hours of delivery

| Characteristic | ≤60s  (n=134) | 61-89s  (n=106) | ≥90s  (n=105) | *P* |
| --- | --- | --- | --- | --- |
| **Within 7 days before delivery** |  |  |  |  |
| WBC (×10^9^/L) | 8.39±2.32 | 8.89±2.44 | 8.69±3.00 | 0.160 |
| NEU (×10^9^/L) | 6.31±2.20 | 6.64±2.30 | 6.54±2.86 | 0.400 |
| LYM (×10^9^/L) | 1.58±0.43 | 1.64±0.44 | 1.59±0.42 | 0.440 |
| EOS (×10^9^/L) | 0.04 (0.02, 0.07) | 0.05 (0.03, 0.08) | 0.04 (0.03, 0.07) | 0.280 |
| BAS (×10^9^/L) | 0.10 (0.10, 0.20) | 0.10 (0.10, 0.20) | 0.20 (0.10, 0.20) | 0.368 |
| MON (×10^9^/L) | 0.44 (0.37, 0.52) | 0.46 (0.37, 0.55) | 0.48 (0.39, 0.56) | 0.210 |
| PLT (×10^9^/L) | 212.14±51.43 | 225.27±52.63 | 208.62±44.41 | 0.086 |
| RBC (×10^12^/L) | 3.97±0.50 | 4.00±0.39 | 3.96±0.32 | 0.770 |
| NEU (%) | 73.81±6.62 | 74.39±6.45 | 74.04±6.41 | 0.760 |
| LYM (%) | 19.75 (15.62, 23.70) | 19.10 (15.62, 23.33) | 19.10 (15.20, 23.60) | 0.740 |
| EOS (%) | 0.04 (0.02, 0.07) | 0.05 (0.03, 0.08) | 0.04 (0.03, 0.07) | 0.560 |
| BAS (%) | 0.20 (0.10, 0.20) | 0.20 (0.10, 0.20) | 0.20 (0.10, 0.30) | 0.590 |
| MON (%) | 5.57±1.54 | 5.36±1.34 | 5.70±1.22 | 0.290 |
| PCV/Hct (%) | 36.36±3.52 | 36.32±4.58 | 36.21±3.30 | 0.730 |
| HGB (g/L) | 118.33±13.01 | 119.77±14.00 | 113.99±24.91 | 0.420 |
| MCV (fL) | 91.31±5.63 | 90.83±10.07 | 90.63±10.28 | 0.980 |
| MCH (pg) | 29.66±2.37 | 30.00±2.79 | 29.54±3.70 | 0.410 |
| MCHC (g/L) | 320.17±40.05 | 323.78±33.40 | 319.68±43.43 | 0.130 |
| RDW (%) | 13.50 (12.93, 14.10) | 13.30 (12.70, 14.30) | 13.20 (12.70, 14.00) | 0.100 |
| PDW (fL) | 16.29±0.93 | 16.30±0.64 | 16.30±0.87 | 0.860 |
| MPV (fL) | 9.88±1.40 | 10.00±1.11 | 10.14±1.35 | 0.540 |
| PCT (%) | 0.21±0.04 | 0.22±0.05 | 0.21±0.04 | 0.038^c^ |
| **Within 24 hours after delivery** | | | | |
| WBC (×10^9^/L) | 10.74±2.35 | 11.38±3.03 | 10.91±2.30 | 0.400 |
| NEU (×10^9^/L) | 8.15±2.10 | 8.70±2.76 | 8.37±2.10 | 0.410 |
| LYM (×10^9^/L) | 1.72 (1.54, 2.05) | 1.80 (1.55, 2.15) | 1.81 (1.58, 2.13) | 0.590 |
| EOS (×10^9^/L) | 0.09 (0.04, 0.13) | 0.09 (0.04, 0.13) | 0.08 (0.05, 0.13) | 0.970 |
| BAS (×10^9^/L) | 0.02 (0.01, 0.03) | 0.02 (0.02, 0.03) | 0.02 (0.02, 0.03) | 0.250 |
| MON (×10^9^/L) | 0.49 (0.43, 0.58) | 0.49 (0.40, 0.62) | 0.48 (0.42, 0.63) | 0.910 |
| PLT (×10^9^/L) | 194.61±46.23 | 199.80±49.88 | 192.89±37.87 | 0.580 |
| RBC (×10^12^/L) | 3.51 (3.31, 3.88) | 3.65 (3.38, 3.89) | 3.57 (3.25, 3.85) | 0.340 |
| NEU (%) | 75.15±7.22 | 74.47±10.40 | 75.27±8.43 | 0.990 |
| LYM (%) | 17.47±4.69 | 17.67±5.77 | 17.49±4.52 | 0.930 |
| EOS (%) | 0.80 (0.40, 1.10) | 0.80 (0.30, 1.28) | 0.70 (0.50, 1.10) | 0.870 |
| BAS (%) | 0.20 (0.10, 0.30) | 0.20 (0.10, 0.30) | 0.20 (0.10, 0.30) | 0.840 |
| MON (%) | 4.70 (4.20, 5.40) | 4.60 (4.00, 5.20) | 4.80 (4.20, 5.70) | 0.093 |
| PCV/Hct (%) | 32.87±6.99 | 33.37±4.67 | 33.43±7.46 | 0.450 |
| HGB (g/L) | 105.42±17.41 | 107.46±19.32 | 104.50±20.58 | 0.550 |
| MCV (fL) | 89.50±11.27 | 88.37±14.69 | 89.12±14.13 | 0.700 |
| MCH (pg) | 29.69±2.33 | 30.00±6.94 | 30.39±5.74 | 0.610 |
| MCHC (g/L) | 322.10±13.06 | 323.40±12.36 | 323.84±12.26 | 0.630 |
| RDW (%) | 13.71±1.27 | 13.67±1.37 | 13.54±1.12 | 0.600 |
| PDW (fL) | 15.91±1.18 | 16.09±0.87 | 15.79±1.44 | 0.840 |
| MPV (fL) | 9.87±1.13 | 9.91±1.09 | 10.16±1.34 | 0.230 |
| PCT (%) | 0.18 (0.16, 0.22) | 0.19 (0.17, 0.22) | 0.19 (0.17, 0.21) | 0.340 |

Abbreviation: IQR, Interquartile Range. SD, Standard Deviation. WBC, White Blood Cell Count. NEU, Neutrophil. LYM, Lymphocyte. EOS, Eosinophil. BAS, Basophils. MON, Monocytes. PLT, Platelet Count. RBC, Red Blood Cell Count. PCV, Packed Cell Volume. HGB, Hemoglobin. MCV, Mean Corpuscular Volume. MCH, Mean Corpuscular Hemoglobin. MCHC, Mean Corpuscular Hemoglobin Concentration. RDW, Red Cell Distribution Width. PDW, Platelet Distribution Width. MPV, Mean Platelet Volume. PCT, Plateletcrit

^c^ The significance values were adjusted for multiple testing by Bonferroni correction and statistically significant differences were found in ≤60s group vs 61-89s group and 61-89s group vs ≥90s group.

**Supplemental table 2** Repeated-measures ANOVA fixed effects for six measurements of blood bilirubin at different cord natural cessation of pulsation time subgroups

|  | Estimate | Std. Error | t | *P* |
| --- | --- | --- | --- | --- |
| (Intercept) | 82.0817 | 5.9431 | 13.811 | ＜0.001 |
| Group | 2.1505 | 2.8474 | 0.755 | 0.450 |
| Time48h | 52.0078 | 6.3706 | 8.164 | ＜0.001 |
| Time72h | 83.3287 | 6.3706 | 13.080 | ＜0.001 |
| Time5d | 97.9478 | 6.3706 | 15.375 | ＜0.001 |
| Time10d | 66.9404 | 6.3706 | 10.508 | ＜0.001 |
| Time42d | -5.9519 | 6.3706 | -0.934 | 0.350 |
| Group:Time48h | 0.2005 | 3.0522 | 0.066 | 0.948 |
| Group:Time72h | 0.7835 | 3.0522 | 0.257 | 0.797 |
| Group:Time5d | 1.4825 | 3.0522 | 0.486 | 0.627 |
| Group:Time10d | 3.7623 | 3.0522 | 1.233 | 0.218 |
| Group;Time42d | -2.1482 | 3.0522 | -0.704 | 0.482 |

**Supplemental table3** Neonatal need for medication or healthcare products at 6 time points in different cord natural cessation pulsation time groups

| Characteristic | ≤60s  (n=134) | 61-89s  (n=106) | ≥90s  (n=105) | *P* |
| --- | --- | --- | --- | --- |
| Time (after birth), (n, %) |  |  |  |  |
| 24 hours |  |  |  | 0.300 |
| Yes | 0 | 0 | 1(1.0) |  |
| No | 134(100.0) | 106(100.0) | 104(99.0) |  |
| 48 hours |  |  |  | ＞0.990 |
| Yes | 3(2.2) | 3(2.8) | 3(2.9) |  |
| No | 131(97.8) | 103(97.2) | 102(97.1) |  |
| 72 hours |  |  |  | 0.510 |
| Yes | 12(9.0) | 6(5.7) | 6(5.7) |  |
| No | 122(91.0) | 100(94.3) | 99(94.3) |  |
| 5 days |  |  |  | 0.590 |
| Yes | 23(17.2) | 14(13.2) | 19(18.1) |  |
| No | 111(82.8) | 92(86.8) | 86(81.9) |  |
| 10 days |  |  |  | 0.600 |
| Yes | 34(25.4) | 23(21.7) | 29(27.6) |  |
| No | 100(74.6) | 83(78.3) | 76(72.4) |  |
| 42 days |  |  |  | 0.320 |
| Yes | 58(43.3) | 36(34.0) | 44(41.9) |  |
| No | 76(56.7) | 70(66.0) | 61(58.1) |  |

**Supplemental table4** Fixed coefficients and tests of different groups and time points of utilization of health products or drugs after delivery of the newborn

| Model term | Coefficient | SE | *t* | *P* | 95%CI | |
| --- | --- | --- | --- | --- | --- | --- |
|  |  |  |  |  | Lower | Upper |
| Intercept | -1.690 | 0.429 | -3.940 | 0.000 | -2.532 | -0.849 |
| ≤60s | -0.000 | 9.233 | -0.000 | 0.001 | 0.393 | 1.577 |
| 61-89s | 0 |  |  |  |  |  |
| 24 hours | -14.144 | 91.947 | -0.154 | 0.878 | -194.504 | 166.215 |
| 48 hours | -3.126 | 0.406 | -7.702 | 0.000 | -3.922 | -2.330 |
| 72 hours | -2.073 | 0.273 | -7.588 | 0.000 | -2.609 | -1.537 |
| 5 days | -1.284 | 0.221 | -5.819 | 0.000 | -1.717 | -0.851 |
| 10 days | -0.738 | 0.199 | -3.700 | 0.000 | -1.129 | -0.347 |
| 42 days | 0 |  |  |  |  |  |
| Intercept | -2.180 | 0.493 | -4.424 | 0.000 | -3.147 | -1.213 |
| 61-89s | 0.897 | 0.338 | 2.652 | 0.008 | 0.233 | 1.561 |
| ≥90s | 0 |  |  |  |  |  |
| 24 hours | -4.913 | 1.013 | -4.848 | 0.000 | -6.901 | -2.925 |
| 48 hours | -3.094 | 0.440 | -7.033 | 0.000 | -3.957 | -2.231 |
| 72 hours | -2.367 | 0.332 | -7.129 | 0.000 | -3.018 | -1.716 |
| 5 days | -1.216 | 0.240 | -5.065 | 0.000 | -1.687 | -0.745 |
| 10 days | -0.651 | 0.217 | -2.999 | 0.003 | -1.076 | -0.225 |
| 42 days | 0 |  |  |  |  |  |
| Intercept | -1.141 | 0.420 | -2.715 | 0.007 | -1.964 | -0.317 |
| ≤60s | 0.534 | 0.291 | 1.837 | 0.066 | -0.036 | 1.104 |
| ≥90s | 0 |  |  |  |  |  |
| 24 hours | -5.161 | 1.011 | -5.105 | 0.000 | -7.145 | -3.178 |
| 48 hours | -3.534 | 0.472 | -7.493 | 0.000 | -4.459 | -2.609 |
| 72 hours | -2.254 | 0.285 | -7.898 | 0.000 | -2.814 | -1.694 |
| 5 days | -1.246 | 0.218 | -5.712 | 0.000 | -1.674 | -0.818 |
| 10 days | -0.742 | 0.200 | -3.699 | 0.000 | -1.135 | -0.348 |
| 42 days | 0 |  |  |  |  |  |
